# Supplementary material for: Fractal dimension of cortical functional connectivity networks & severity of disorders of consciousness
Source: PLoS One. 2020 Feb 13;15(2):e0223812. doi: 10.1371/journal.pone.0223812 (PMC7017993; doi:10.1371/journal.pone.0223812)
Supplement: Attachment — Submitted filename: plos_point_by_point_final.pdf [file pone.0223812.s003.pdf]

We would like to thank the reviewers for taking the time to read and think critically about our manuscript. Below, find our point-by-point response (original reviewer comments in standard font, *our discussion in italics).*

On behalf of all the authors,
~Thomas Varley

Journal Requirements

1. *We have updated our material to be complaint with the PloS Style Guide.*

2. *We have included brief captions for both supplementary datasets.*

3. *We have added that written consent was obtained for the Control volunteers, and that written consent was given by the legal representatives of the DOC patients – this is consistent with requirements for approval by the relevant oversight bodies mentioned in the text.*

4. *We are pleased that the editors have found our other work. We refrained from self-citation as the linked paper is still undergoing peer-review and has not been published yet. At the request of the editors, we have added a brief discussion in our Discussion section:*

> *The results presented here are consistent with previous work of ours using similar techniques on fMRI data from adult volunteers under the influence of LSD and psilocybin (Varley et al., 2019). There we found that both LSD and psilocybin significantly increased the fractal dimension of high-threshold FC networks, and LSD increased the Higuchi fractal dimension of BOLD signals. In the current study, a decrease in temporal and spatial fractal dimension is associated with loss of consciousness, while in the previous case, an increase in fractal dimension is associated with a subjective increase in the "complexity" or vividness of conscious experience. Taken together, these complementary results provide converging lines of evidence that temporal and spatial fractal dimension are meaningfully related to a range of conscious states and that such analyses is feasible using BOLD signals.*

5. We have addressed the question of data sharing in the revised cover letter, as directed.

Reviewer 1:

1.1 It is not mathematically elegant the way in which the matrix M is defined on p. 11. The value of M_{ij} is defined in terms of itself. It makes sense in a programming setting where M is a variable whose value changes. If the authors want to explain successive filters that were applied to M, they can introduce M', M'' and then M, or something like that. Finally, "All surviving values M_{ij} < 0 ...". Is it correct? If I understood the procedure, at that moment all values were 0 or 1.

This is an excellent point – to simplify matters, we have defined M_{ij} as the correlation matrix, and then described removing the diagonals without further matrix notation, and then define the binary,

thresholded matrix as M_{ij}'. We hope that this is more elegant. We also explicitly state that M_{ij}' is a sparse, symmetric, binary matrix.

1.2 With respect to the use of "null graphs" or "null networks", it can be ambiguous at it can refer to graphs with no edges. Please indicate that you refer to a "null model" or graphs not representing brain connectivity.

To address this point, the following section has been added to the "Formation of Null Graphs" subsection:

> *"Here, "null graph" refers to a network with the same number of nodes but a structure unrelated to brain connectivity. By creating these null networks, we can explore the general behavior of the network fractal dimension algorithm and use this knowledge to inform our empirical findings."*

Reviewer 2:

2.1. More relevant and related studies should be cited and compared in the paper regarding fractal analysis applied to consciousness, and fractal analysis of brain networks. For example, in the second paragraph of page 4, references Ieva 2014 and Ieva 2015 are very generic. HFD studies on consciousness at the end of Introduction (page 5) are relative old. Please, cite, comment and compare current studies on FD and consciousness such as Ruiz de Miras et al. 2019 and other references in that study: "Fractal dimension analysis of states of consciousness and unconsciousness using transcranial magnetic stimulation". Computer Methods and Programs in Biomedicine 175, pp. 129-137. 2019.

Thank you for these sources: the study by Ruiz de Miras et al., is a fascinating read. To address this concern and bring our discussion up-to-date with the literature, we have added the following:

> *"Research has found that the fractal dimension of brain activity is related to the level and content of consciousness: the fractal dimension of EEG signals reliably falls during sleep (Pereda et al., 1998, Klonowski et al., 2005, Klonowski et al., 2010, Ruiz de Miras et al., 2019), sedation (Ferentes et al., 2006), and loss of consciousness following the administration of anesthsia (Klonowski et al., 2010, Spasic et al., 2011, Ruiz de Miras et al., 2019) . Temporal fractal dimension also changes during internally vs. externally generated perceptions (Ibanez-Molina et al., 2014, Bornas et al., 2013) and during altered states such as hypnosis (Solhjoo et al., 2005). In addition to analysis of the temporal domain, Ruiz de Miras et al., (2019) also explored the fractal dimension of the spatial distribution of significant sources following TMS-perturbation and a spatiotemporal measure of the same. Similar to the temporal measures, spatiotemporal fractal dimension also dropped when consciousness was lost under conditions of propofol anesthesia and NREM sleep.*

2.2. Page 6. "To quantify the fractal dimension …, the Compact Box Burning (CBB) algorithm was used…". Please, explain the reasons why that method for computing the FD of networks was selected,

and include an appropriate reference to the paper describing the method. Please, clarify whether the author used any third-party software for computing the CBB or a home-made program.

We have added the following portion to the "Calculating Network Fractal Dimension" section:

> *We chose the CBB algorithm because it is an easily-implemented algorithm that can handle smaller networks than alternatives such as the maximum-excluded mass-burning algorithm (Song et al., 2007). We used a modified implementation of the freely-available code from https://hmakse.ccny.cuny.edu/software-and-data/.}*

2.3. Page 11. Definitions of F(t) and Hi(t) are missing.

This has been corrected.

2.4. Page 14. Table 1 would provide more information if it includes which comparisons have a p-value < 0.05

We have added comparison indicators to the table.

2.5. Page 13. Statistical analysis. It is a bit strange for me that authors have used Python for statistical analysis instead of using SPSS or MATLAB as usual in the field. Please, explain.

The Python language provides robust, well-established infrastructure for statistical analysis through the SciPy Stats packages. As all our other analyses were done in Python (using packages like Numpy, NetworkX, etc), it was natural to continue in this vein. The primary author is also most familiar with the Python language.

2.6. Page 15. 3.3. Higuchi temporal fractal dimension. Authors claim that they did not include the HFD analysis on awake subjects because of the different and smaller number of samples regarding DOC subjects. However, this reviewer thinks that this analysis should be included, explaining the possible limiting factors.

Following this suggestion, we included all the Awake HFD analyses, as well as the truncated results, see Results Section 3.3: Higuchi Fractal Dimension.

2.7. Page 15. "Surprisingly, we found no significant correlation between temporal fractal dimension and network fractal dimension …". It is not such a surprise, since related studies, such as Ruiz de Miras et al. 2019, are in the same line. HFD needs be complemented with other measures to adequately characterize the signal.

In light of this advice, we have added the following to the Discussion:

> *These results point to several possible subsequent investigations. First, how do temporal and spatial fractal dimensions relate to other temporal (e.g. Lempel-Ziv complexity, sample entropy, etc) and spatial or network measures (scale-freeness, small-worldness, etc)? Previous work on discriminating states consciousness has indicated that multiple measures working in concert work*

> *better than any single unidimensional scaler (Kim et al., 2018, Ruiz de Miras et al., 2019) , as different measures describe different aspects of the system. Interrogating how different temporal and spatial measures interact may provide insight both into the nature of consciousness and the behaviour of these formalisms.*

Reviewer 3:

In this research report entitled "Fractal dimension of cortical functional connectivity networks predicts severity in disorders of consciousness" the authors explored the FD of functional connectivity networks and time series of BOLD signals from healthy participants and patients with disorders of consciousness (MCS, VS). They found that in all measures the FD for healthy participants was higher than for patients; and it was higher for MCS than VS patients. These results might indicate that the FD of functional structures of the brain is sensitive to abnormal states of consciousness.

General comment
The structure of the paper is convenient and it is clearly developed along the manuscript. The theoretical background is also clear and simple to explain but in my opinion some confusion exists on the use of terms as fractal dimension, fractal character and complexity. I will raise this issue in the next paragraph section.
The study is well described and its predictions are straightforward. However, I do not think it is the best experimental design. Although the results are very easy to interpret, I believe additional analyses would provide more information about the nature of the differences between the FD in the experimental groups. I will comment this point in the section about the results.

Comments on theoretical concepts
1- One of the more repeated concepts in the manuscript is 'complexity'. I believe that in general we need to take care when use it because in most studies it is a synonym of 'randomness'. In this study complexity is identified with a system between order and randomness, with being more or less fractal or with the level of FD that a given signal exhibits. From my point of view, complexity is not a well defined concept in science. Even in the so called science of complexity there is no agreement about the exact meaning of it. Hence, the authors need to define what complexity is in the framework of this particular investigation. I encourage the authors to give a specific working definition for complexity in the introduction section and follow it in the rest of the manuscript.

We thank the reviewer for pointing out this omission. We have added discussion to the introduction briefly discussing the limitations of randomness-based definitions of complexity (specifically, the commonly used metric Lempel-Ziv compressibility) and have attempted to take the suggestion and more explicitly state what we mean when we refer to "complexity":

> *"Complexity" is operationalized in different ways in different fields, but a common theme is to use "randomness" (often measured in terms of incompressibility) as a proxy measure for a more nebulous concept. In the study of consciousness, Lempel-Ziv compressibility (LZ_{C}) (Ziv and*

Lempel, 1978) is the most frequently-used measure (Zhang et al., 2001, Scharter et al., 2015, Schartner et al., 2017, Schartner et al., 2017, Wang et al., 2017) In LZ_{C} analyses a maximally random (or incompressible) signal would be indicated as having the highest "complexity." This is, however, a somewhat counter-intuitive understanding of what we mean when we talk about the "complexity" of brain activity: the brain is complex not because it is highly random, but because it combines an incredible degree of order with a high degree of unpredictability. It is hard to imagine how a brain outputting algorithmically random noise could doing anything at all, let alone supporting vivid consciousness and cognition. Not only is the brain both structured and unpredictable, it also shows one of the hallmarks of complex systems writ large: emergent dynamics over multiple scales. With this in mind, we strongly feel that consciousness science requires further discussion and refinement of what "complexity" means in the context of the brain. As a working definition, we propose that complexity should be understood as a fundamentally multi-scale phenomena, emerging in systems that display both a high degree of emergent structure and organization as well incompressible and unpredictable features.

2- Another confusing point, at least for me, is that in this work it is suggested that one of the goals is to study if the functional networks are fractal-like (goal 1 in page 5). One might investigate this particular question by looking the small world properties or power law characteristics, etc.; but I believe that the FD alone does not indicate if a given object or network is fractal. It is suggested here that if the FD is higher for a given network it would show more fractal properties than other network that exhibits less FD. This is not necessarily true. The definition of a fractal is any structure made of copies of itself (self- similar) and depending of the nature of its self-similarity the FD will be high or low. For example, The Koch curve has a FD of 1.26, the Sierpinski triangle has a FD of 1.58, and I would not say that the latter is more fractal than the former. We might also use an empirical example: it is very likely to obtain a very high Higuchi's fractal dimension for a random signal, and it does not mean that this is self affine or fractal. In general, FD indicates the density of the structure being measured, or the roughness of the object. It would be possible that in a given context a high FD indicate that it is a fractal and I believe that this is what it needs to be clarified in the manuscript. It would be needed to justify why a higher FD for a functional network means that it is a fractal. I know that in theory any topological dimension that is fractional belongs to a fractal structure but this is just for mathematical objects. Natural objects can show fractional dimensions calculated with algorithms of approximation without being fractal-like. For example, if you use the Higuchi's fractal dimension with a simple sinusoidal curve, the FD would be slightly higher than 1. And this signal is not a fractal indeed.

The question of how to interpret the change in the fractal dimension is an interesting one, and admittedly has been inadequately addressed by much of the literature. In response to the reviewers comments, we have changed the wording throughout the paper to describe just differences in the magnitude of the fractal dimension, instead of comparing whether a given network or time-series is "more" or "less" fractal. We have also added the following to the discussion section:

> *"One difficulty of much of this work is creating an intuitive understanding of what it means for a system to have a "lower" fractal dimension than another. Considerable previous research has shown that loss of consciousness is associated with lower fractal dimensions (Pereda et al., 1998, Klonowski et al., 2005, Ferentes et al., 2006, Klonowski et al., 2010, Spasic et al., 2011, Ruiz de Mira et al., 2019), but what does that mean? Different fractal shapes may have higher or lower fractal dimensions, but that doesn't necessarily mean that they are "more" or "less" fractal. In the context of a box-counting analysis, where $d_{B} \propto -ln(N_{B}(l_{B})) / ln(l_{B})$, a higher fractal dimension corresponds to a steeper slope: small changes in l_{B} correspond to comparatively more dramatic changes in $N(l_{B})$. This may indicate a "rougher" topology, with a more heterogenous distribution of high- and low-density regions. Ruiz de Mira et al., (2019) discuss fractal dimension in terms of both integration and differentiation, suggesting that alterations to the fractal dimension may represent differences in the ability of a system to balance these competing properties."*

3- I believe that the relationship between consciousness and complexity is not very clear in the manuscript. Complexity is related in this work with criticality as well as with the concept of complexity developed in Tononi's theory. This is quite confusing and difficult to understand by a reader who is not familiar with these two perspectives. I would define the theoretical framework with more precision: A) Is this study testing differentiation and integration as a marker of consciousness? In this case, complexity is very well defined and one might discuss the results in this context. B) Is this study directed to find fractal structures and relate them with conscious states? Then I would try to link complexity with fractal dimension, as I suggested above, and give a solid argument to conceive FD as an indicator of self-affinity.

To make the connection between fractal dimension and complexity more clear we have made several changes. The first is we have reduced our references to Integrated Information Theory: we had not intended that to be our primary framework, preferring instead to focus on the Entropic Brain Hypothesis (which avoids some of IITs more radical claims). The EBH is more focused on the relationship between consciousness and critical dynamics, which provides the framing for our study: one of the hallmarks of criticality is the emergence of fractal structures and so we attempted to test the EBH by looking to see if changes to fractal topologies and dynamics were associated to changes (in this case reduction in) level of consciousness. We have attempted to make this much more explicit.

Comments on the methodology and the FD measures used in the study

1. A specific question I would like to raise here is Do authors really address the goal one of the study introduced in page 5? In order to state that networks constructed suppressing weak edges, have a fractal character, it would be convenient to obtain a different type of functional network and show that it is not fractal (or it is less fractal). Authors could have obtained networks with weak edges and use them for comparison. Moreover, this design would be also convenient to show if the FD decreases only in the networks with fractal characteristics vs networks with less fractal structure.

We thank the reviewer for this insight into the paper. While we agree the further analyses will definitely provide insight into the relationships between all of these measures and level of consciousness, we feel that they would be outside the purview of this particular study. We have added discussion of these next steps to our discussion section:

> *“These results point to several possible subsequent investigations. First, how do temporal and spatial fractal dimensions relate to other temporal (e.g. Lempel-Ziv complexity, sample entropy, etc) and network (scale-freeness, small-worldness, etc)? Previous studies have used power-law, or heavy-tailed degree distributions as an indicator of fractal structure (Basset et al., 2006) and associated changes to the scaling exponent with alterations to consciousness (Liu et al., 2014). While the degree distribution and box-counting describe different aspects of the network structure (Kim et al., 2007), the relationships between them seem like a fruitful area to explore. Of particular interest is how different network topologies facilitate the propagation of information, which may give insight into how changes to brain structure and connectivity alter information processing and integration. Similarly, previous work on discriminating states consciousness has indicated that multiple measures working in concert work better than any single unidimensional scaler (Kim et al., 2018, Ruiz de Miras et al., 2019) , as different measures describe different aspects of the system. Interrogating how different temporal and spatial measures interact may provide insight both into the nature of consciousness and the behaviour of these formalisms.”*

2. In this section I also want to point out that I am a bit confused about the values of the measures obtained in the study. There are three different measures. The first one is the FD of the Networks (NFD); the second one is the FD of the Adjacency matrix (AdFD); and the third one is the Higuchi’s FD (HFD).
In principle one might expect that a network maximum value of NFD would be 3 just because the maximal density of an object in a three dimensional space is 3. However, obtained values are higher than 3. Because I am not an expert in the box counting algorithm presented here, I might be wrong in this point.
The values of the AdFD measures are between one and two and this is reasonable because one might expect that FD=2 will be the more compact object in this space. But the values of HFD are a bit weird because they should range between 1 (straight line) and 2 (a very dense signal filling the entire 2 dimensional space). The means reported here are below 1. I believe it might be due to the selection of the k parameter but the authors need to check if there is an error in the estimation.

In regards to the network fractal dimension being >3, this is not as unusual as it might appear at first glance: while the reviewer is certainly correct that a physically instantiated network must “fit” into a 3-dimensional space at maximum, the functional-connectivity network, being a network of statistical correlations is not physically embedded in the same way a structural network might be (in the context of networks, dimensionality is something of a tricky thing). For context, Song et al., (cited in this paper) found a fractal dimension of ~3.5 for an e. coli intracellular network and a fractal dimension of ~4.1 for the world-wide web, showing that abstract networks do not necessarily have to follow an intuitive sense of embedding in space.

We concur about the oddness of the values of HFD (we scratched our heads about this too), and have added a discussion of this to the results section, acknowledging this. The results are reasonably robust to parameter selection (as noted, we searched a fairly wide parameter space, following established methods). We suspect it may be in part an artifact arising from the combination of short time-series with very low frequencies (BOLD time-series are comparatively very slow).

3. It would be useful to include the characteristics of the BOLD signals used in the study. At least authors should report the length of the segments, and the AD rate at which they were registered. Does 'samples' in section 3.3 refer to number of points in a segment? Is there only one segment per participant?

We have reported the number of TRs (this is the number of samples) in each scan and made it more explicit what we are referring to when we discuss the "length" of a scan.

Comments on the results
The non parametric statistical approach introduced by the authors is adequate. The results showed that the FD was different for each group and measure. However, I would not say that one might predict (as suggested in the title of the manuscript) the severity of the disorder of consciousness. I think that in order to predict it would be necessary to include a larger sample of participants as well as a mathematical model of prediction (ROC curves, linear or non linear multiple regression, or a more sophisticated classifier). Hence I would say that the discussion should consider this fact and a different verb should be used in the title.

This is a good point. We have changed the title and removed "predictive validity" from the discussion section, replacing it with:

> *"a single, easily digestible measure that seems to be relevant in a clinically meaningful population."*
